# Synthesis of neodymium ferrite incorporated graphitic carbonitride (NdFe_2_O_4_@g-C_3_N_4_) and its application in the photodegradation of ciprofloxacin and ampicillin in a water system

**DOI:** 10.1039/d2ra08070b

**Published:** 2023-02-13

**Authors:** Adewale Adewuyi, Rotimi A. Oderinde

**Affiliations:** a Department of Chemical Sciences, Faculty of Natural Sciences, Redeemer's University Ede Osun State Nigeria walexy62@yahoo.com +2348035826679; b Department of Chemistry, Faculty of Science, University of Ibadan Ibadan Oyo State Nigeria; c Department of Chemistry, University of Cambridge Lensfield Road Cambridge CB2 1EW UK

## Abstract

Purification of antibiotic-contaminated drinking water sources is faced with limitations. Therefore, this study incorporated neodymium ferrite (NdFe_2_O_4_) in graphitic carbonitride (g-C_3_N_4_) to form NdFe_2_O_4_@g-C_3_N_4_ as a photocatalyst for removing ciprofloxacin (CIP) and ampicillin (AMP) from aqueous systems. X-ray diffraction (XRD) revealed a crystallite size of 25.15 nm for NdFe_2_O_4_ and 28.49 nm for NdFe_2_O_4_@g-C_3_N_4_. The bandgap is 2.10 and 1.98 eV for NdFe_2_O_4_ and NdFe_2_O_4_@g-C_3_N_4_, respectively. The transmission electron micrograph (TEM) images of NdFe_2_O_4_ and NdFe_2_O_4_@g-C_3_N_4_ gave an average particle size of 14.10 nm and 18.23 nm, respectively. Scanning electron micrograph (SEM) images showed heterogeneous surfaces with irregular-sized particles suggesting agglomeration at the surfaces. NdFe_2_O_4_@g-C_3_N_4_ (100.00 ± 0.00% for CIP and 96.80 ± 0.80% for AMP) exhibited better photodegradation efficiency towards CIP and AMP than NdFe_2_O_4_ (78.45 ± 0.80% for CIP and 68.25 ± 0.60% for AMP) in a process described by pseudo-first-order kinetics. NdFe_2_O_4_@g-C_3_N_4_ showed a stable regeneration capacity towards degradation of CIP and AMP with a capacity that is above 95% even at the 15th cycle of treatment. The use of NdFe_2_O_4_@g-C_3_N_4_ in this study revealed its potential as a promising photocatalyst for removing CIP and AMP in water systems.

## Abstract 2

Neodymium ferrite incorporated graphitic carbonitride (NdFe_2_O_4_@g-C_3_N_4_) was synthesized. NdFe_2_O_4_@g-C_3_N_4_ completely removed ciprofloxacin and ampicillin from contaminated water. NdFe_2_O_4_@g-C_3_N_4_ exhibited outstanding regeneration capacity for reuse.

## Graphical abstract

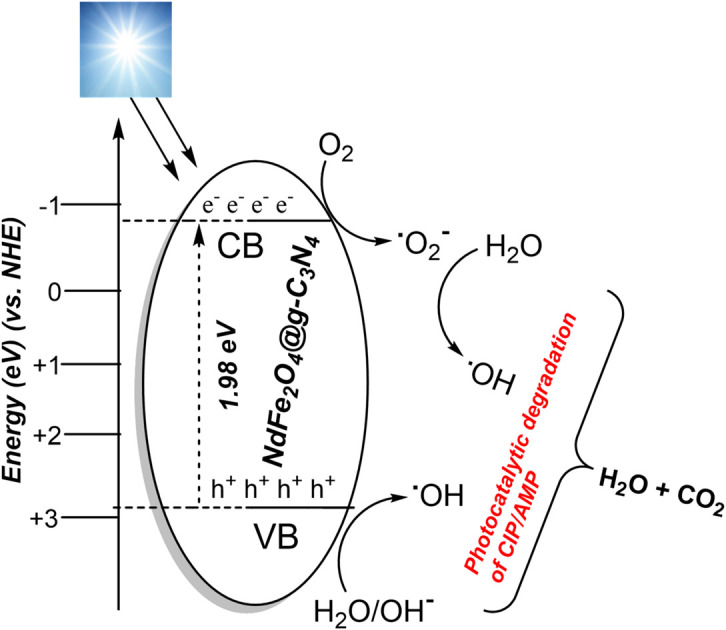

## 1. Introduction

The provision of clean drinking water is challenged with contamination from emerging and unexpected pollutants.^1,2^ The unexpected contaminants in water are of different forms, most of which are classified as unregulated pollutants in water systems. Among the unregulated contaminants in water, antibiotics are of serious concern. They have been transferred by leaching into environmental water systems such as surface and underground water systems, which can serve as drinking water sources.^3^ Authors have detected antibiotics in different drinking water sources,^4–6^ and due to the current challenge of incomplete removal of antibiotics in treated drinking water sources, antibiotic contaminated water sources have become worrisome. A recent study has reported the presence of antibiotics in bottled water and other drinking water sold on the market.^7,8^ Unfortunately, when antibiotics are exposed to environmental water systems, they can become metamorphosed into new forms that are more dangerous than the primary antibiotics. The harmful metabolites or metamorphoses of the antibiotics may threaten human health as they can cause diseases.^9,10^ Survival of antibiotics in environmental water systems has been attributed to the emergence of antibiotic-resistance strains of microorganisms,^10,11^ which compromises the efficacy of the antibiotics. It is essential to develop means of completely removing antibiotics from drinking water sources, which is the focus of this study.

Ciprofloxacin (CIP) and ampicillin (AMP) are common antibiotics found in drinking water sources (surface and underground water systems). CIP was detected in Baghdad City, Iraq's water treatment plant.^7^ Moreover, a high level of CIP has been reported from groundwater in South India,^12^ while AMP was reported in water samples collected from the treatment plant's secondary effluent drinking water, river, groundwater, and lagoon water.^13^ Recently, a study revealed high frequency (64.5% strains) of resistance to AMP in the Białka River discharge treatment resource.^14^ Such contamination with AMP has a significant environmental impact that cannot be overlooked. Moreover, discharge from such treatment plants may enter surface and underground water systems if not properly managed. Another study has shown that veterinary use is one of the significant contributors of antibiotics to environmental water systems resulting in the contamination of drinking water sources.^15^ Even though they exist in trace amounts, they negatively impact the environment and human beings. Antibiotics can adsorb solid substances in the environment and become transported over an extended range of distances. They have varying photostability and water solubility and are susceptible to biodegradation which may transform them into eco-unfriendly products.^16,17^ This study focuses on CIP and AMP as antibiotics of interest because of their prevalence and their frequent occurrence in drinking water sources globally. They are commonly found in drinking water sources because they are affordable and can be purchased from local pharmacy stores without prescription from physicians, especially in developing countries. They are among the most frequently used antibiotics for diseases treatment with inevitable presence in environmental water system used as drinking water sources. They have been reported across the globe in environmental drinking water sources. Therefore, it is important that an efficient method is developed to remove CIP and AMP completely from water system before they cause any havoc.

Most antibiotics used in animal and human treatment may become metabolized to different degrees, and when excreted, they remain unchanged in their active forms.^14^ Although the amount is too low to impose potency against pathogenic organisms, they affect the pathogens' genetic identity, creating identity resistance against known antibiotics which is a serious public health problem.^18,19^ The continuous introduction of antibiotics to the environment has led to a severe threat to public health, which has been of global concern. It has necessitated the need to develop an efficient technique for removing antibiotics such as CIP and AMP from the water system before they get into drinking water sources or during water treatment. Since CIP and AMP are common in drinking water sources, this study shall focus on developing an efficient technique for their removal in a water system. This is essential to avoid the danger that the presence of CIP and AMP may cause in water.

Several approaches have been used to remove CIP and AMP in water systems, but most have limitations. Among these limitations are high process costs and incomplete removal of CIP and AMP during treatment. Therefore, this study proposes using a nanomaterial composite as a photocatalyst for removing CIP and AMP in water. Nanocomposites have played an essential role as photocatalysts for degrading organic molecules.^20^ The multi-component nature of nanocomposite gives it an advantage for functionality enhancement over single-component material for photocatalysis. Ferrites are an example of multi-component materials with the possibility of being photocatalytic. The unique properties of ferrites, such as small size, thermal stability, simple synthesis route, magnetism, photosensitivity, and conductivity, give it application in water treatment. Although many photocatalysts have been reported but most of them are only active in the ultraviolet (UV) region of the light spectrum. Therefore, it becomes necessary to provide a UV light source for them to be active since UV light is not abundantly available in nature. Unfortunately, the provision of UV light source is an additional cost which increases the cost of photocatalysis process. Fortunately, ferrite such as NdFe_2_O_4_ is expected to be active in the visible light region, which is advantageous for photocatalytic process. A previous study has reported the use of 3% CdS QDs/CaFe_2_O_4_@ZnFe_2_O_4_ as visible light active photocatalyst for the degradation of norfloxacin.^21^ Tetracycline and CIP have been reportedly degraded with the use of p-CaFe_2_O_4_@n-ZnFe_2_O_4_ which is a visible light active photocatalyst, however, the degradation is less than 100%.^22^ Since visible light is abundant in nature and freely available at no cost, using NdFe_2_O_4_ as a visible light active photocatalyst for the degradation of antibiotics (CIP and AMP) is beneficial because it reduces the cost of photocatalysis process. Although different ferrites have been reported in water treatment,^23–25^ there is a little report on neodymium ferrite (NdFe_2_O_4_). Nd is a reactive lanthanide with excellent properties that may improve the performance of iron oxide when made into ferrite.

NdFe_2_O_4_ is chosen in this study because of its photosensitivity and ideal size, which qualifies it as a photocatalyst. Moreover, it is stable and can be cheaply produced. Unfortunately, the particles can agglomerate because of their size, which reduces their efficiency. To overcome the challenge of agglomeration, NdFe_2_O_4_ may be incorporated into graphitic carbon nitride (g-C_3_N_4_) to form NdFe_2_O_4_@g-C_3_N_4_. The g-C_3_N_4_ serves as a carbon source to stabilize the particles of NdFe_2_O_4_, preventing agglomeration. Furthermore, g-C_3_N_4_ is a carbon-based metal-free semiconductor catalyst that may help boost the catalytic performance of NdFe_2_O_4_. It has a graphite-like layered structure with high chemical and thermal stability, outstanding electronic properties and is non-toxic.^26–29^ Polymeric g-C_3_N_4_ possess good photocatalytic property for the degradation of organic pollutants under visible light irradiation.^30–33^ Similar concept of using carbon source material have been previously reported using ZnFe_2_O_4_-carbon allotropes for the photodegradation of norfloxacin^34^ and ZnFe_2_O_4_@RGO for the degradation of CIP.^35^ This study proposes a co-catalytic performance of NdFe_2_O_4_ and g-C_3_N_4_ in a single entity as NdFe_2_O_4_@g-C_3_N_4_. With the continuous and frequent detection of CIP and AMP in environmental drinking water sources and their incomplete removal during water treatment, this study aims to develop an efficient means for their complete removal in an aqueous system. The study proposes using NdFe_2_O_4_@g-C_3_N_4_ as a photocatalyst for removing CIP and AMP in water. From our understanding, there is scanty information on the synthesis and use of NdFe_2_O_4_@g-C_3_N_4_ as a catalyst for the photodegradation of CIP and AMP in a water system.

## 2. Experimental data

### 2.1. Materials

Sodium hydroxide (NaOH), neodymium(iii) chloride hexahydrate (NdCl_3_·6H_2_O), iron(iii) chloride hexahydrate (FeCl_3_·6H_2_O), polyvinylpyrrolidone, nitric acid (HNO_3_), melamine, ethanol (C_2_H_5_OH), chloroform (CH), ammonium oxalate (AO), hydrochloric acid (HCl), AMP, CIP, isopropyl alcohol (IPA), and other chemicals were ordered from Aldrich Chemical Co., England.

### 2.2. Synthesis of NdFe_2_O_4_ particles

Solutions of NdCl_3_·6H_2_O (0.2 M) and FeCl_3_·6H_2_O (0.4 M) were mixed in a 1 L beaker for 60 min in the presence of polyvinylpyrrolidone (50 mg) while stirring continuously at 80 °C. The solution pH (10–12) was maintained by adding NaOH (2 M) dropwise until precipitate appeared while stirring continued for 2 h. The solution was cooled to room temperature, filtered, and washed severally with C_2_H_5_OH and deionized water until the filtrate was neutral to litmus. The residue was dried at 105 °C in the oven for 5 h and transferred to the furnace at 550 °C for 18 h.

### 2.3. Preparation of g-C_3_N_4_

Briefly, melamine (10.00 g) was weight into a crucible and covered appropriately. The covered crucible and its content were transferred into a muffle furnace and heated to 550 °C at a controlled heating rate of 5 °C min^−1^ for 3 h. After cooling to room temperature, the content of the crucible was poured into HNO_3_ (0.5 M) in a 250 mL conical flask and stirred for 1 h. The solution was centrifuged (4200 rcf, 10 min) three times while washing with deionized water. The g-C_3_N_4_ obtained was dried at 105 °C for 5 h in the oven.

### 2.4. Preparation of NdFe_2_O_4_@g-C_3_N_4_ particles

NdFe_2_O_4_ (2.00 g) and g-C_3_N_4_ (2.00 g) were separately dispersed in deionized water (20.00 mL) in a 100 mL beaker and sonicated for 1 h. Both mixtures were combined and further sonicated for another 1 h. The mixture was transferred to an autoclave and hydrothermally treated (200 °C, 6 h). It was cooled to room temperature and centrifuged (4200 rcf, 10 min) twice while washing with deionized water. The solid mass obtained was dried at 105 °C for 3 h and annealed in a muffle furnace at 400 °C (at 1 °C min^−1^) for 2 h.

### 2.5. Characterization of NdFe_2_O_4_ and NdFe_2_O_4_@g-C_3_N_4_ particles

NdFe_2_O_4_ and NdFe_2_O_4_@g-C_3_N_4_ were analyzed for their functional group composition using Fourier transformed infrared spectroscopy (FTIR, PerkinElmer, RXI 83303, USA) with spectrum recorded at 400–4500 cm^−1^. The diffraction pattern was taken on an X-ray diffractometer (2*θ*) at 5–90° having filtered Cu Kβ radiation (40 kV and 40 mA). The thermogravimetric analysis (TGA) was achieved on TGA/DSC 2 Star^e^ system (DB V1300A-ICTA-Star^e^), while the UV-visible absorption was read on UV-visible spectrophotometer. The TEM images (Talos F200X G2) and SEM images (JEOL JSM-5510LV) were taken for morphology while the elemental composition was confirmed using energy-dispersive X-ray spectroscopy (EDS) (INCA mics EDX system).

### 2.6. Photocatalytic degradation of CIP and AMP by NdFe_2_O_4_ and NdFe_2_O_4_@g-C_3_N_4_

The photodegradation evaluation of CIP and AMP by NdFe_2_O_4_ and NdFe_2_O_4_@g-C_3_N_4_ was achieved in the presence of visible light using a solar simulator (Xe, 150 W) possessing filter holder.^25^ Test solutions (50 mL) of CIP (5.00 mg L^−1^) and AMP (5.00 mg L^−1^) were separately exposed to simulated visible light irradiation in the presence of NdFe_2_O_4_ (0.1 g) or NdFe_2_O_4_@g-C_3_N_4_ (0.1 g) in a beaker (100 mL) while stirring continuously at 120 rpm for 180 min. The UV lamp and the test solution were kept 20 cm from each other. Samples were withdrawn at an interval from the test solution to monitor the degradation of CIP or AMP using a UV-visible spectrophotometer (PerkinElmer, Lambda). Photodegradation was confirmed by UV-visible measurements taken at the appropriate wavelength as follows; CIP (*λ*_max_ = 271 nm) and AMP (*λ*_max_ = 420 nm). The impact of process parameters, such as the effect of weight on the photodegradation of CIP and AMP, was determined by varying the weight (0.1 to 0.5 g) of NdFe_2_O_4_@g-C_3_N_4_ while the effect of pH was determined by varying the test solutions pH from 2–10. The effect of test solution concentration on the photodegradation process was estimated by varying the concentration of CIP and AMP from 1.00 to 5.00 mg L^−1^. A dark experiment was conducted to establish the adsorption–desorption equilibrium to check whether adsorption was taking place along with the photodegradation process. All experimental conditions (concentration, pH, weight, and time) were kept constant during the dark experiment except exposure to visible light (no irradiation). All the experiments were repeated three times, and values were presented as a mean. The degradation efficiency was calculated as follows:1
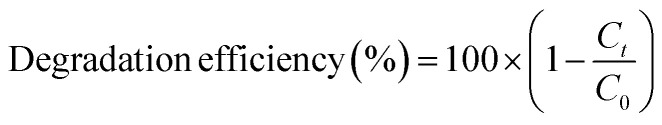
where *C*_0_ is the initial concentration of the test solution and *C*_*t*_ is the concentration of the test solution at time *t*. For the dark experiment, the adsorption capacity (*q*_e_) and the percentage removal (% removal) expressed towards CIP and AMP were calculated as follows:2
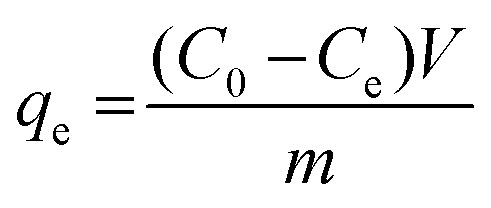
3
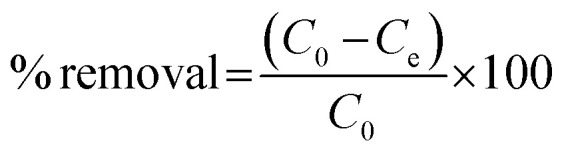
where *C*_0_ (mg L^−1^) represents the initial concentration of the test solution, *C*_e_ (mg L^−1^) is the test solution concentration at equilibrium; the solution volume is presented as *V* (in litre), the weight (g) of NdFe_2_O_4_ or NdFe_2_O_4_@g-C_3_N_4_ is given as *m*, and the adsorption capacity is given as *q*_e_ (mg g^−1^).

### 2.7. Scavenging of reactive oxygen species

The mechanism of action for the photodegradation of CIP and AMP using NdFe_2_O_4_@g-C_3_N_4_ was investigated by evaluating the role of reactive oxygen species (ROS) in the photodegradation process. This was achieved by examining the role of isopropyl alcohol (IPA) as a hydroxyl radical (OH˙) scavenger, ammonium oxalate (AO) as a scavenger for a hole (h^+^), and chloroform (CH) as a scavenger for superoxide ion radical (˙O_2_^−^). Each scavenger (1 mM) was included in the test solution while carrying out the photodegradation process. The process parameters such as weight (0.1 g), pH (7.5), time (180 min), and test solution concentration (5.00 mg L^−1^) for the photodegradation (with and without scavengers) were kept constant.

### 2.8. Regeneration for reuse and stability of NdFe_2_O_4_@g-C_3_N_4_

At the end of the photodegradation process, NdFe_2_O_4_@g-C_3_N_4_ was recovered from the solution by filtration and washed with solvent (deionized water, 0.1 M HCl, C_2_H_5_OH or mixture of C_2_H_5_OH and 0.1 M HCl (3 : 2)) to regenerate it for reuse. It was oven dried at 105 °C for 5 h before being reused for the photodegradation of CIP or AMP. The treated test solution was subjected to inductively coupled plasma optical emission spectroscopy (ICP-OES) analysis to check whether there was elemental leaching of NdFe_2_O_4_@g-C_3_N_4_ into the treated test solution during the photodegradation process. Analysis with ICP-OES was conducted at the end of each treatment cycle with NdFe_2_O_4_@g-C_3_N_4_. Furthermore, FTIR analysis was conducted at the end of the treatment cycle on the test solution to check for the presence of organic molecules. The photostability of NdFe_2_O_4_@g-C_3_N_4_ for the photodegradation of CIP and AMP was evaluated in fifteen (15) successive cycles of operation. At the end of the 15th cycle, the NdFe_2_O_4_@g-C_3_N_4_ was analyzed for XRD and FTIR to check for changes in the structural identity of NdFe_2_O_4_@g-C_3_N_4_.

## 3. Results and discussion

### 3.1. Synthesis and characterization of NdFe_2_O_4_ and NdFe_2_O_4_@g-C_3_N_4_

**Fig. 1:**
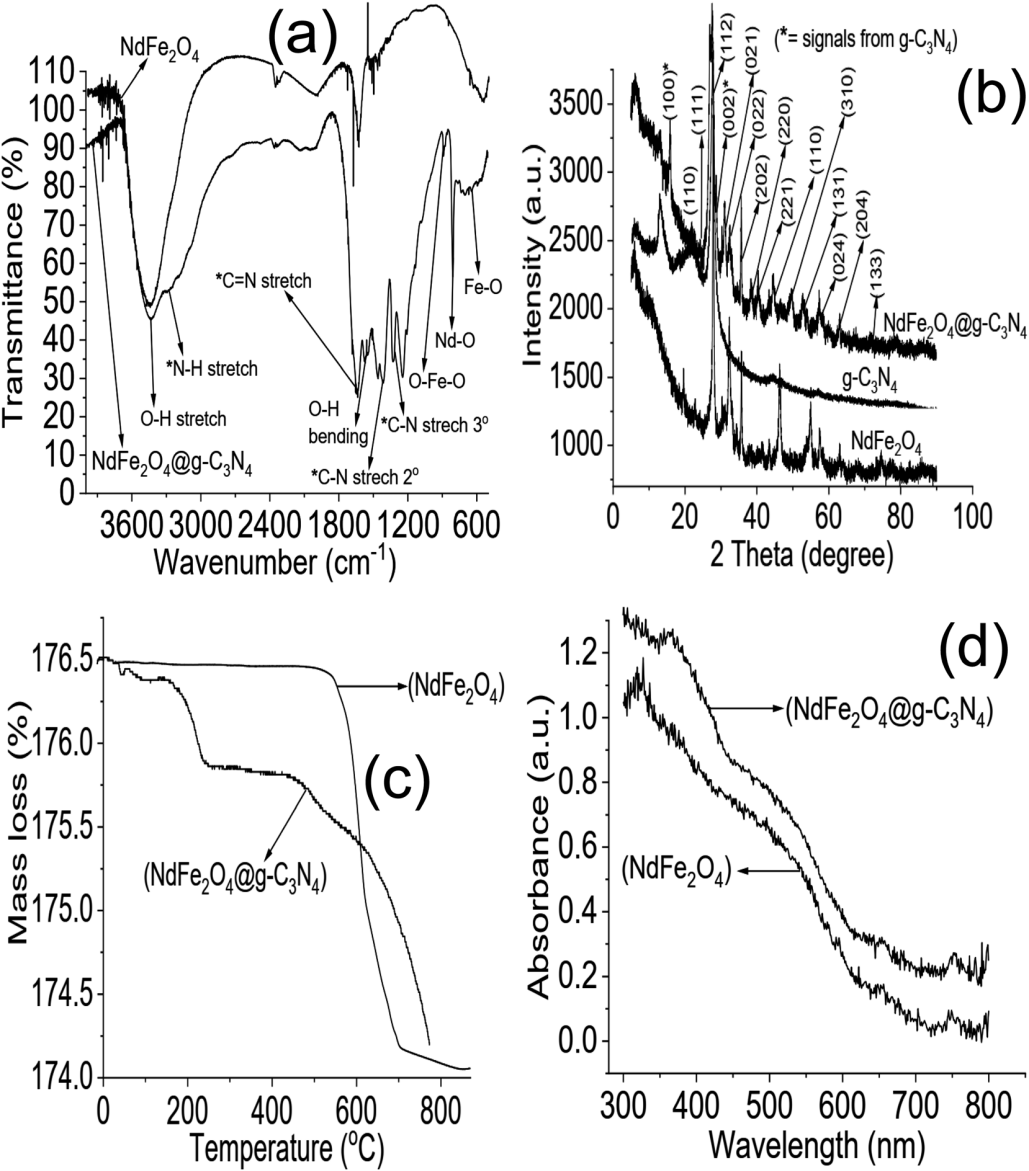
FTIR (a), XRD (b), TGA (c) and UV-visible spectra (d) of NdFe_2_O_4_ and NdFe_2_O_4_@g-C_3_N_4_.

The FTIR results of NdFe_2_O_4_ and NdFe_2_O_4_@g-C_3_N_4_ are presented in Fig. 1a. The signal at 3421 cm^−1^ in both NdFe_2_O_4_ and NdFe_2_O_4_@g-C_3_N_4_ may be attributed to the O–H stretch of adsorbed water molecules while the signal at 3413 cm^−1^ which corresponds to N–H stretch was only found in NdFe_2_O_4_@g-C_3_N_4_. The C=N stretch at 1632 cm^−1^ only appeared in NdFe_2_O_4_@g-C_3_N_4_, while the O–H bending at 1621 cm^−1^ was found in both NdFe_2_O_4_ and NdFe_2_O_4_@g-C_3_N_4_. The signal at 1332 and 1234 cm^−1^ are characteristic of aromatic C–N stretch, which may be associated with secondary and tertiary amine fragments, respectively.^36,37^ The O–Fe–O stretch was seen at 1140 cm^−1^ in both NdFe_2_O_4_ and NdFe_2_O_4_@g-C_3_N_4_. The 720 and 622 cm^−1^ signals were attributed to Nd–O and Fe–O vibrations in NdFe_2_O_4_ and NdFe_2_O_4_@g-C_3_N_4_, respectively.

The X-ray diffractions patterns for NdFe_2_O_4_, g-C_3_N_4_ and NdFe_2_O_4_@g-C_3_N_4_ are shown in Fig. 1b with patterns that correspond to (002), (021), (022), (024), (100), (110), (111), (112), (131), (133), (202), (204), (220), (221) and (310) with the most intense peak appearing at 2*θ* = 27.87°. The peaks corresponding to (021), (022), (024), (111), (112), (131), (133), (202), (204), (220), (221) and (310) confirms the synthesis of NdFe_2_O_4_. The pattern aligned with powder diffraction file (PDF) no. 00-074-1473 and no. 65-318.^38,39^ The signals asterisked (*), mainly (002) and (100), were signals emanating from g-C_3_N_4_, which confirmed the inclusion of g-C_3_N_4_ in NdFe_2_O_4_ to form NdFe_2_O_4_@g-C_3_N_4_. Their crystallite sizes of NdFe_2_O_4_ and NdFe_2_O_4_@g-C_3_N_4_ were calculated from the broadening line of their reflections at (112) according to the Debye–Scherrer's formula:^40^4
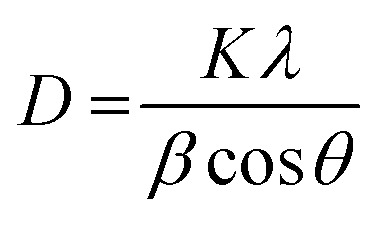
from eqn (4), *D* represents the average crystallite size of NdFe_2_O_4_ or NdFe_2_O_4_@g-C_3_N_4_, *K* is a constant (0.89), the X-ray wavelength is given as *λ* (1.5406 Å), *β* is the entire width of diffraction line, and *θ* is the Bragg's angle taken at peak (112) which describes the patter of NdFe_2_O_4_ and NdFe_2_O_4_@g-C_3_N_4_.^41^ The crystallite size exhibited by NdFe_2_O_4_ is 25.15 nm, while that of NdFe_2_O_4_@g-C_3_N_4_ is 28.49 nm. The crystallite size obtained for NdFe_2_O_4_ is lower than that of NdFe_2_O_4_@g-C_3_N_4_, which may be due to the inclusion of g-C_3_N_4_ in the structure of NdFe_2_O_4_ to form NdFe_2_O_4_@g-C_3_N_4_. The presence of g-C_3_N_4_ may have resulted in an extended bulk crystallite size. Precisely, this concept may help describe the diffusion property of NdFe_2_O_4_ and NdFe_2_O_4_@g-C_3_N_4_ as previously expressed:^42,43^

5 *τ* = *r*^2^π^2^*D*

the average diffusion time to the surface of NdFe_2_O_4_ and NdFe_2_O_4_@g-C_3_N_4_ is *τ* while *D* is the diffusion coefficient. The diffusion time becomes longer when the crystallite size is large, which makes the particles susceptible to aggregation or recombination when used as a catalyst. In the occurrence of aggregation or recombination, the catalytic performance becomes reduced.^43,44^ Invariably, it is paramount that the crystallite size is small for the best catalytic performance.^45^ Comparatively, the crystallite size exhibited by NdFe_2_O_4_ and NdFe_2_O_4_@g-C_3_N_4_ is smaller than the range of crystallite sizes (37 to 45 nm) reported previously for spinel ferrite^46^ suggesting NdFe_2_O_4_ and NdFe_2_O_4_@g-C_3_N_4_ as materials with catalytic potentials.

The TGA results for the thermal stability of NdFe_2_O_4_ and NdFe_2_O_4_@g-C_3_N_4_ are shown in Fig. 1c. The spectra revealed three major mass losses in NdFe_2_O_4_ and five significant mass loses in NdFe_2_O_4_@g-C_3_N_4_. The mass loss at 60 to 155 °C in NdFe_2_O_4_ and NdFe_2_O_4_@g-C_3_N_4_ (first loss) may be attributed to the loss of adsorbed molecules like H_2_O, CO_2_, and other volatile molecules. The observed loss from 155 to 700 °C in NdFe_2_O_4_, which is the second loss, may be attributed to mass loss due to dehydration of the OH group in its structure, involving intra and intermolecular reactions and formation of metal oxides.^47,48^ Mass loss from above 700 °C in NdFe_2_O_4_ (third loss) may be attributed to phase change. Furthermore, the mass loss from 150 to 200 °C (second loss) and 200 to 380 °C (third loss) in NdFe_2_O_4_@g-C_3_N_4_ may be assigned to the decomposition of Fe(OH)_3_ to FeOOH and FeOOH to γ-Fe_2_O_3_, respectively as previously reported.^38^ The mass loss from 380 to 600 °C (fourth loss) in NdFe_2_O_4_@g-C_3_N_4_ was attributed to the decomposition of the g-C_3_N_4_ structure.^49^ There is a gradual stable change in mass from 600 to 800 °C in NdFe_2_O_4_@g-C_3_N_4_ which may be due to the gradual decomposition of g-C_3_N_4_ structure to N_2_, (CN)_2_, NH_3_ and HCN.^50,51^Fig. 1d revealed the response of NdFe_2_O_4_ and NdFe_2_O_4_@g-C_3_N_4_ to visible light absorption. The spectra showed the absorption region for NdFe_2_O_4_ and NdFe_2_O_4_@g-C_3_N_4_ in the visible light area, which suggest them as potential material with activity within the visible light region. This activity is the reason for proposing the materials for photocatalytic degradation of CIP and AMP. Since these materials are active in the visible light region, their use as photocatalysts will not incur an additional cost since visible light is readily available at no cost, unlike in the case of materials that are only photoactive in the UV region which requires additional cost of incurring UV light source.

**Fig. 2:**
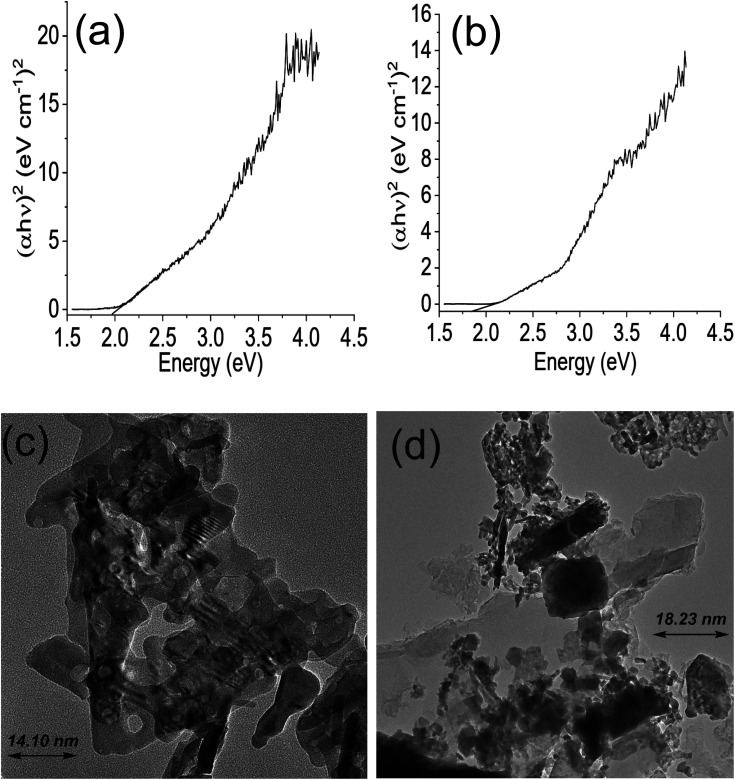
Tauc plot for NdFe_2_O_4_ (a), Tauc plot for NdFe_2_O_4_@g-C_3_N_4_ (b), TEM of NdFe_2_O_4_ (c) and TEM of NdFe_2_O_4_@g-C_3_N_4_ (d).

**Fig. 3:**
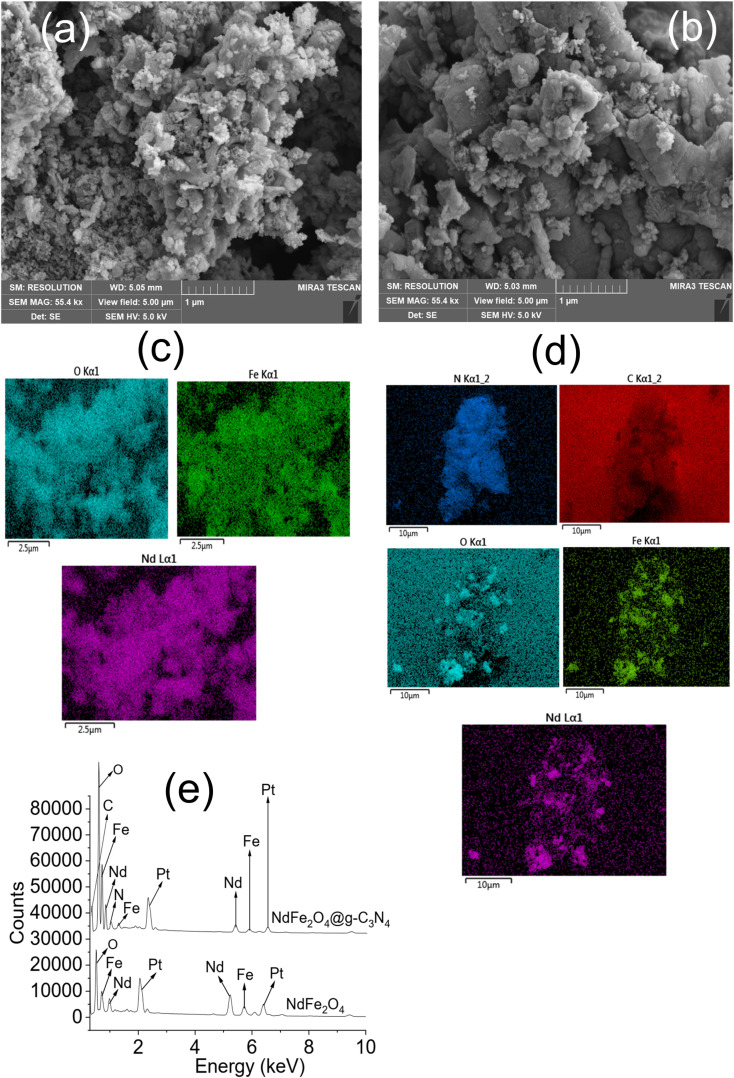
SEM of NdFe_2_O_4_ (a) SEM of NdFe_2_O_4_@g-C_3_N_4_ (b), elemental mapping of NdFe_2_O_4_ (c), elemental mapping of NdFe_2_O_4_@g-C_3_N_4_ (d) and EDS of NdFe_2_O_4_ and NdFe_2_O_4_@g-C_3_N_4_ (e).

The Tauc plot for NdFe_2_O_4_ and NdFe_2_O_4_@g-C_3_N_4_ are shown in Fig. 2a and b, respectively. Their optical band gap was described as follows:

6 (*αhv*)^2^ = *A*(*hv* − *E*_g_)

where *hv* is the frequency of the irradiated light, *A* represents the proportionality constant, *E*_g_ is the bandgap, and *α* is the absorption coefficient. The bandgap is 2.10 and 1.98 eV for NdFe_2_O_4_ and NdFe_2_O_4_@g-C_3_N_4_, respectively. There was a reduction in the bandgap when g-C_3_N_4_ was included in the structure of NdFe_2_O_4_ to produce NdFe_2_O_4_@g-C_3_N_4_. The bandgap reduction indicates better absorption of visible light by NdFe_2_O_4_@g-C_3_N_4_ for enhanced photocatalytic performance. The TEM images of NdFe_2_O_4_ (Fig. 2c) and NdFe_2_O_4_@g-C_3_N_4_ (Fig. 2d) revealed an average particle size of 14.10 nm and 18.23 nm, respectively. The particles are oval-shaped and arranged in a consistent regular pattern. The surface morphology revealed from SEM images showed the surfaces of NdFe_2_O_4_ (Fig. 3a) and NdFe_2_O_4_@g-C_3_N_4_ (Fig. 3b) to be heterogeneous, with irregular-sized particles suggesting agglomeration at the surfaces. The particles are stacked, but the surface of NdFe_2_O_4_ appeared grainier than that of NdFe_2_O_4_@g-C_3_N_4_, which may be due to the larger crystallite size of NdFe_2_O_4_@g-C_3_N_4_. The elemental mapping of NdFe_2_O_4_ (Fig. 3c) and NdFe_2_O_4_@g-C_3_N_4_ (Fig. 3d) revealed the presence of Nd, Fe and O in both NdFe_2_O_4_ and NdFe_2_O_4_@g-C_3_N_4_ whereas C and N were additionally seen in NdFe_2_O_4_@g-C_3_N_4_. The EDS results (Fig. 3e) showed peaks corresponding to the elemental composition of NdFe_2_O_4_ and NdFe_2_O_4_@g-C_3_N_4_, which further corroborates the elemental mapping.

### 3.2. Photodegradation study

**Fig. 4:**
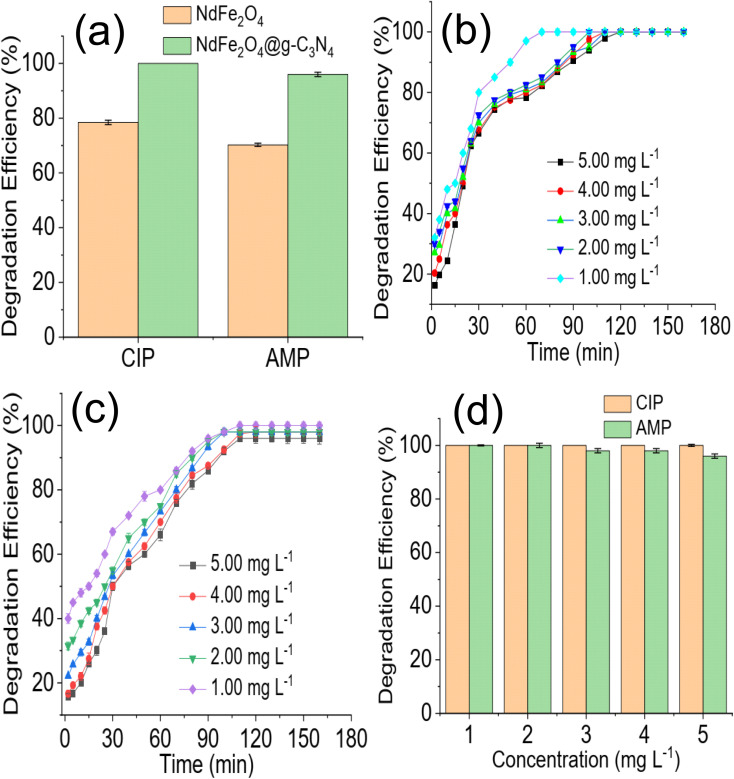
Comparison of the preliminary degradation efficiency expressed by NdFe_2_O_4_ and NdFe_2_O_4_@g-C_3_N_4_ towards CIP and AMP (a), time dependent degradation of CIP in the presence of NdFe_2_O_4_@g-C_3_N_4_ at different concentration (b), time dependent degradation of AMP in the presence of NdFe_2_O_4_@g-C_3_N_4_ at different concentration (c) and effect of solution concentration on the degradation of CIP and AMP in the presence of NdFe_2_O_4_@g-C_3_N_4_ (d).

A preliminary investigation of the degradation efficiency of NdFe_2_O_4_ and NdFe_2_O_4_@g-C_3_N_4_ (Fig. 4a) revealed a better performance by NdFe_2_O_4_@g-C_3_N_4_. NdFe_2_O_4_ had efficiencies of 78.45 ± 0.80 and 68.25 ± 0.60% for the photodegradation of CIP and AMP, respectively. These degradation efficiencies increased to 100.00 ± 0.00 and 96.80 ± 0.80% for the degradation of CIP and AMP, respectively, when NdFe_2_O_4_@g-C_3_N_4_ was used. It showed that NdFe_2_O_4_@g-C_3_N_4_ has superior performance over NdFe_2_O_4_ for the photodegradation of CIP and AMP. A previous study revealed that increasing crystallite size has strong influence on enhancing photodegradation process.^52^ In this present study the superior performance of NdFe_2_O_4_@g-C_3_N_4_ may be attributed to its higher crystallite size when compared with NdFe_2_O_4_. Therefore, further, and subsequent investigation of the degradation of CIP and AMP in this study was conducted using NdFe_2_O_4_@g-C_3_N_4_ alone. The time degradation of CIP in the presence of NdFe_2_O_4_@g-C_3_N_4_ is shown in Fig. 4b. The degradation was progressive over time until it reached equilibrium. However, the initial degradation capacity was highest for the lowest concentration (1.00 mg L^−1^), which may be because the amount of species of CIP in the test solution at the study concentration (1.00 mg L^−1^) was lower than the other concentrations studied and were (CIP species in solution) quickly and rapidly degraded within the first few periods of the degradation process. Furthermore, the 100% degradation of CIP was achieved at the 80 min of degradation process when the concentration was 1.00 mg L^−1^ whereas complete degradation was attained at 120 min when the concentration was 5.00 mg L^−1^. A similar observation was obtained for AMP (Fig. 4c). Furthermore, a 100% degradation of AMP was only attained at the lowest concentration (1.00 mg L^−1^) studied. As the concentration of the test solution increased, degradation efficiency dropped. For example, at the highest concentration (5.00 mg L^−1^) investigated for AMP, the degradation efficiency obtained is 96 ± 0.80%, whereas, at the least concentration (1.00 mg L^−1^) studied, the degradation efficiency is 100%. This observation is clearly described in Fig. 4d, which shows the decrease in degradation efficiency expressed towards AMP as concentration increased from 1.00 to 5.00 mg L^−1^. This observation may be attributed to the fact that as concentration increased, more species of the antibiotics were available in the solution requiring more capacity of NdFe_2_O_4_@g-C_3_N_4_ for the degradation process.

**Fig. 5:**
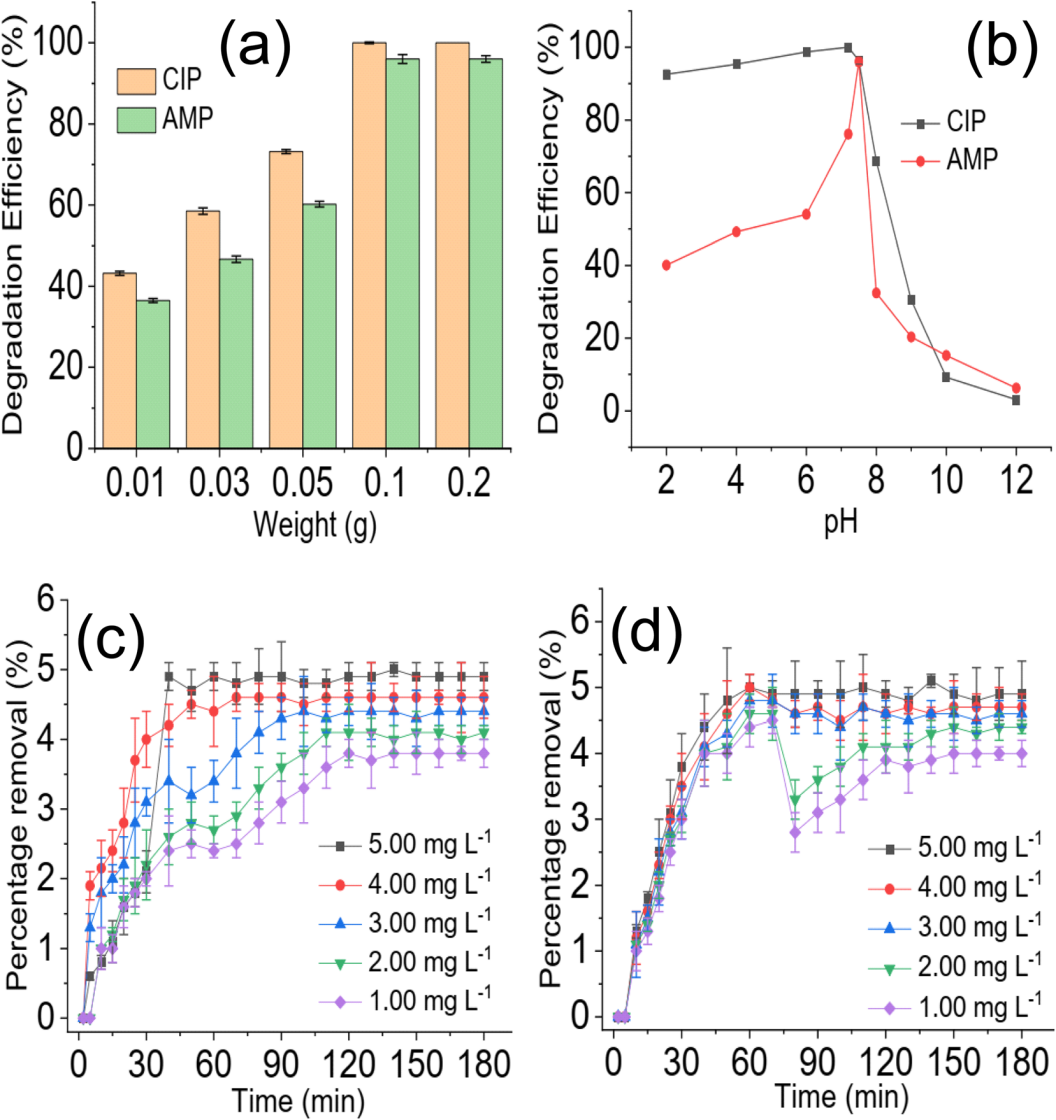
Effect of NdFe_2_O_4_@g-C_3_N_4_ weight on the degradation of CIP and AMP (a), effect of solution pH on the degradation of CIP and AMP (b), percentage removal for the sorption of CIP (c) and AMP (d) by NdFe_2_O_4_@g-C_3_N_4_ in the dark experiment.

The effect of NdFe_2_O_4_@g-C_3_N_4_ weight on the degradation process revealed that increasing the weight of NdFe_2_O_4_@g-C_3_N_4_ from 0.01 to 0.2 favoured the degradation process as the capacity of NdFe_2_O_4_@g-C_3_N_4_ to degrade CIP and AMP was enhanced with increase in weight, which may be attributed to the increase in active surface area for photodegradation process. Interestingly, similar degradation efficiency was obtained for 0.1 and 0.2 g weights of NdFe_2_O_4_@g-C_3_N_4_. However, increasing the weight of NdFe_2_O_4_@g-C_3_N_4_ above 0.2 g resulted in a decrease in the performance of NdFe_2_O_4_@g-C_3_N_4_. This observation may be because as weight increased beyond 0.2 g, a lesser amount of visible light was able to penetrate the solution for effective photoactivation of NdFe_2_O_4_@g-C_3_N_4_, such may result in a shielding effect which obstructs the excitation of NdFe_2_O_4_@g-C_3_N_4_ due to excessive scattering of the photon; similar observation has been reported previously.^53^ The test solution pH of CIP and AMP were varied from 2 to 12 while maintaining a concentration of 5.00 mg L^−1^, weight of 0.1 g and degradation time of 180 min to investigate the effect of pH on the degradation efficiency of NdFe_2_O_4_@g-C_3_N_4_. Result obtained is presented in Fig. 5b. The degradation efficiency of NdFe_2_O_4_@g-C_3_N_4_ increased as pH increased towards pH 7. NdFe_2_O_4_@g-C_3_N_4_ performed better as pH tends towards neutral, suggesting that more ROS were generated in the test solution at this pH for the degradation process. The performance of NdFe_2_O_4_@g-C_3_N_4_ decreased as pH tends towards alkaline pH, suggesting a decrease in the amount of ROS generated. A dark experiment was conducted to understand whether adsorption significantly affected the photodegradation of CIP and AMP by NdFe_2_O_4_@g-C_3_N_4_. The percentage removal expressed towards CIP is 4.90 ± 0.20% (Fig. 5c), while that of AMP is 4.90 ± 0.50% (Fig. 5d). The percentage removal for the dark experiment increased with time with an adsorption capacity of 2.45 mg g^−1^ expressed by NdFe_2_O_4_@g-C_3_N_4_ towards CIP and AMP. Both photocatalysis and adsorption took place at the same time. However, the contribution from adsorption is about 5% of the total performance demonstrated by NdFe_2_O_4_@g-C_3_N_4_ for removing CIP and AMP from the solution, suggesting that about 95% of the contribution made by NdFe_2_O_4_@g-C_3_N_4_ towards the removal of CIP and AMP from solution was *via* photocatalysis.

**Fig. 6:**
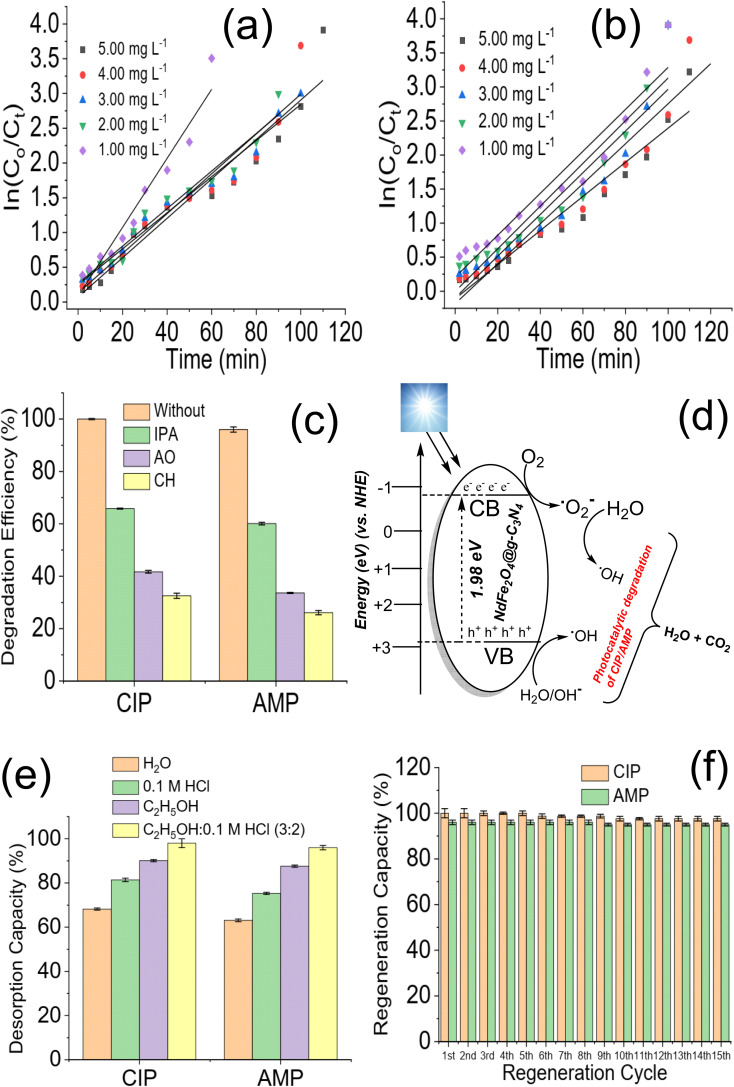
Plot of ln *C*_0_/*C*_*t*_*versus* irradiation time for the degradation of CIP (a) and AMP (b) at different solution concentrations in the presence of NdFe_2_O_4_@g-C_3_N_4_, degradation efficiency of NdFe_2_O_4_@g-C_3_N_4_ towards CIP and AMP with and without ROS scavengers (c), proposed mechanism for the photodegradation of CIP, and AMP (d), desorption efficiency of NdFe_2_O_4_@g-C_3_N_4_ after washing with different solvent systems (e) and regeneration capacity of NdFe_2_O_4_@g-C_3_N_4_ expressed towards CIP and AMP at different treatment cycle (f).

Data generated from the photodegradation were fitted for the pseudo-first-order kinetic model to better understand the photodegradation process of CIP and AMP using NdFe_2_O_4_@g-C_3_N_4_, which may be expressed as:7
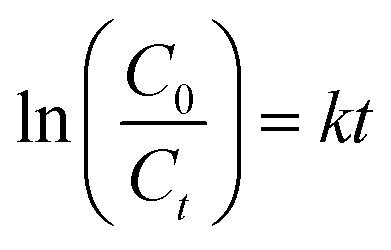
where *C*_0_ and *C*_*t*_ are the initial concentrations of CIP and AMP and concentrations of CIP and AMP at a time “*t*”, respectively, *K* is the pseudo-first-order rate constant obtainable from the slope of the plot of ln *C*_0_/*C*_*t*_*versus* time and *t* is the visible light irradiation time. The plot of ln *C*_0_/*C*_*t*_*versus* irradiation time at the different concentrations of CIP and AMP are shown in Fig. 6a and b. The rate constant obtained for the degradation of CIP at different concentrations increased with a decrease in concentration (5.00 mg L^−1^ = 0.0284 min^−1^, 4.00 mg L^−1^ = 0.0286 min^−1^, 3.00 mg L^−1^ = 0.0290 min^−1^, 2.00 mg L^−1^ = 0.031 min^−1^ and 1.00 mg L^−1^ = 0.0497 min^−1^). This suggests that the lower the concentration, the faster the degradation process exhibited by NdFe_2_O_4_@g-C_3_N_4_. Similar result was obtained for AMP (5.00 mg L^−1^ = 0.0251 min^−1^, 4.00 mg L^−1^ = 0.0294 min^−1^, 3.00 mg L^−1^ = 0.0306 min^−1^, 2.00 mg L^−1^ = 0.0340 min^−1^ and 1.00 mg L^−1^ = 0.0380 min^−1^). This observation further corroborates the observation in Fig. 4b and c that as concentration reduces, the initial degradation efficiency increases and the highest efficiency was attained with a decrease in the concentration of CIP and AMP.

### 3.3. Proposed photodegradation mechanism of CIP and AMP

Photodegradation of organic molecules may be linked to the generation of ROS,^53^ which necessitates investigating ROS's role in the photodegradation exhibited by NdFe_2_O_4_@g-C_3_N_4_ towards the removal of CIP and AMP from solution. In order to understand the mechanism of the photodegradation process, the photodegradation of CIP and AMP by NdFe_2_O_4_@g-C_3_N_4_ was conducted in the presence of AO (as a scavenger for the hole (h^+^)), IPA (as hydroxyl radical scavenger) and CH (as a scavenger for superoxide ion radical) as previously mentioned.^25,54^ After 180 min of photodegradation, it became obvious that AO, IPA, and CH played an important role in the photodegradation of CIP and AMP. The role played by the studied scavengers (Fig. 6c) on the degradation process for CIP is in the order CH (32.50 ± 1.0%) > OA (41.70 ± 0.50%) > IPA (65.80 ± 0.20%) while that of AMP is in the order CH (26.10 ± 0.80%) > OA (33.60 ± 0.20%) > IPA (60.10 ± 0.50%). Therefore, the lower the degradation efficiency obtained, the more the impact of the scavenger on the process. It means that the ROS scavenged has more effect on the degradation process. The highest reduction in degradation efficiency of NdFe_2_O_4_@g-C_3_N_4_ towards CIP and AMP was obtained when CH was introduced into the process, suggesting that superoxide ion radical played the most crucial role in the degradation process. It shows that when CH was introduced into the test solution, the superoxide ions in the test solution were scavenged, leading to a reduction in the degradation process. The proposed mechanism for the photodegradation of CIP and AMP by NdFe_2_O_4_@g-C_3_N_4_ involving ˙OH, h^+^, and ˙O_2_^−^ (Fig. 6d) suggest the *in situ* generations of h^+^ and e^−^ from the valence band (VB) and conduction band (CB), respectively, when visible light is absorbed by NdFe_2_O_4_@g-C_3_N_4_. The generated h^+^ reacts with water molecules to produce the H^+^ and ˙OH while the e^−^ further generates the ˙O_2_^−^ from the O_2_. Usually, the h^+^ and e^−^ recombine after a period, and the degradation process ends. However, if the recombination of h^+^ and e^−^ is avoided, the degradation process will continue. To prevent the recombination of h^+^ and e^−^ generated *in situ* by NdFe_2_O_4_@g-C_3_N_4_, g-C_3_N_4_ was incorporated into NdFe_2_O_4_ to serve as a carbon source as well as a co-catalyst. Previous studies have reported a similar concept using carbon dots.^53,55,56^ This study used g-C_3_N_4_ because it is cheaper to produce, and it is also a catalyst. Therefore, during the photodegradation process, g-C_3_N_4_ can serve as an acceptor for trapping h^+^ and e^−^. Once h^+^ and e^−^ are trapped by g-C_3_N_4_, they are fixed at a point which prevents them from interacting. This way, they (h^+^ and e^−^) keep generating ROS for a long time to continue the photodegradation process without recombining prematurely.^53,57,58^ A recent study demonstrated a plausible mechanism by photoluminescence spectroscopy using terephthalic acid as a probing reagent for monitoring ˙OH generation. The study revealed the oxidation mechanism for phenol to take place *via* a Z-scheme unlike the conventional charge-transfer mechanism.^59^ During the Z-scheme, e^−^ are excited to the CB leaving a h^+^ in the VB. The excited e^−^ further migrates to fill corresponding h^+^, which may reduce H_2_O to H_2_O_2_. Furthermore, the h^+^ may independently oxidize the phenol to CO_2_ and H_2_O. The photocatalytic degradation of tetracycline and CIP by p-CaFe_2_O_4_@n-ZnFe_2_O_4_ heterojunction has been described to be vis electron–hole interaction,^22^ which is similar to the mechanism exhibited in the current study. Furthermore, the mechanism of degradation reported for the degradation of CIP using carbon dot embedded ZnO^53^ and Mn/Co composite^60^ are similar to the mechanism of ROS generation *via* electro–hole interaction described for the degradation of AMP and CIP by NdFe_2_O_4_@g-C_3_N_4_.

### 3.4. Regeneration for reuse and stability of NdFe_2_O_4_@g-C_3_N_4_

The ability to successfully reuse NdFe_2_O_4_@g-C_3_N_4_ for a substantial cycle of treatment without losing functionality is vital. This ability contributes to economic viability and marketability. Therefore, the stability of NdFe_2_O_4_@g-C_3_N_4_ for the degradation of CIP and AMP was checked by solvent regeneration for reuse in fifteen cycles. The most suitable solvent for regenerating NdFe_2_O_4_@g-C_3_N_4_ was determined as shown in Fig. 6e. Mixture of C_2_H_5_OH : 0.1 M HCl (3 : 2) exhibited better desorption of CIP (98.00 ± 2.00%) and AMP (96 ± 1.00%) from the surface of NdFe_2_O_4_@g-C_3_N_4_ than the other solvents used. The regeneration of NdFe_2_O_4_@g-C_3_N_4_ after each treatment cycle was achieved using C_2_H_5_OH : 0.1 M HCl. The regeneration capacity of NdFe_2_O_4_@g-C_3_N_4_ for reuse is stable (Fig. 6f). NdFe_2_O_4_@g-C_3_N_4_ was stable towards CIP until the 6th cycle of treatment when its capacity dropped from 100.00 ± 1.00% to 98.70 ± 1.00% and at the 10th cycle where it dropped to 97.60 ± 1.00% and remained stable all through the treatment cycles. However, stability expressed towards AMP was stable until the 9th cycle, where it dropped from 96 ± 1.00% to 95 ± 0.60% and remained steady until the 15th cycle of treatment. The stability of NdFe_2_O_4_@g-C_3_N_4_ for the photodegradation of CIP and AMP is stable even up to the 15th cycle at a regeneration capacity above 95%, suggesting NdFe_2_O_4_@g-C_3_N_4_ to be a promising photocatalyst for the degradation of antibiotics in water treatment.

**Fig. 7:**
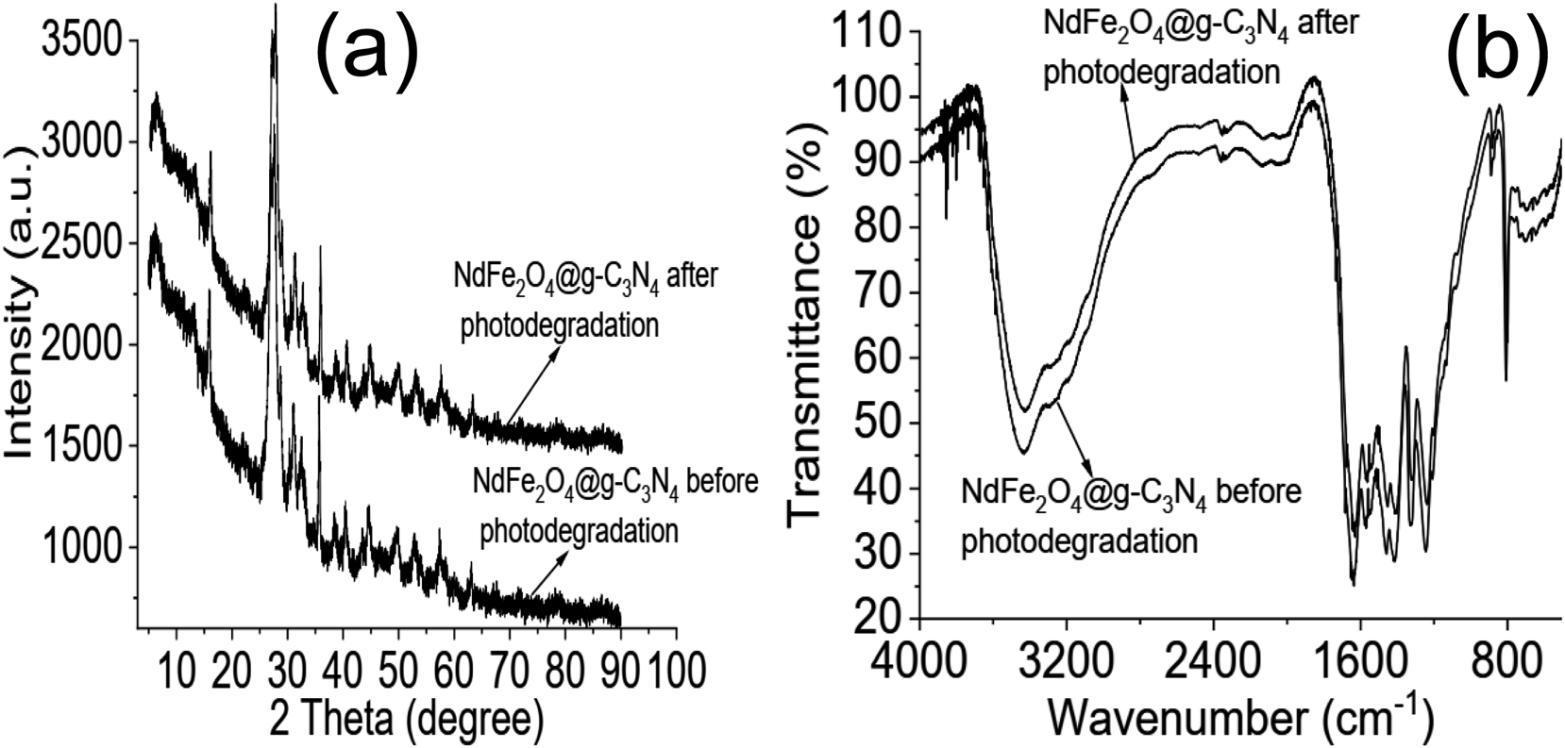
XRD of NdFe_2_O_4_@g-C_3_N_4_ before photodegradation and at 15th cycle of photodegradation (a) and FTIR of NdFe_2_O_4_@g-C_3_N_4_ before photodegradation and at 15th cycle of photodegradation (b).

The stability of NdFe_2_O_4_@g-C_3_N_4_ was further checked after the 15th cycle by subjecting it to FTIR and XRD analysis. Fig. 7 revealed that NdFe_2_O_4_@g-C_3_N_4_ remains structurally stable even after the 15th cycle of treatment. After the ICP-OES analysis, Nd and Fe were not detected in the treated water. On the other hand, the FTIR did not detect the presence of any organic molecules in the treated water samples for CIP and AMP. Although the degradation efficiency attained for AMP is 96.80 ± 0.80%, the FTIR did not indicate presence of organic molecule in the sample which may be due to low concentration of organic molecules present and being out of the detection limit or sensitivity of the FTIR instrument. Results obtained from the current study were compared with previously reported photocatalysts for the degradation of CIP and AMP, as shown in Table 1. The degradation efficiency exhibited by NdFe_2_O_4_@g-C_3_N_4_ for the degradation of CIP is higher than values recently reported for BiVO_4_,^61^ Cu_2_O/MoS_2_/rGO,^62^ TiO_2_/SnO_2_ composite^63^ and CeO_2_/g-C_3_N_4_.^64^ The regeneration capacity expressed by NdFe_2_O_4_@g-C_3_N_4_ also compares favourably with other photocatalysts. Only ZnO^65^ demonstrated similar degradation efficiency to NdFe_2_O_4_@g-C_3_N_4_. However, using ZnO requires a UV source which is an additional process cost that may increase the cost of photodegradation. A previous study using MWCNTs-CuNiFe_2_O_4_ exhibited better results than NdFe_2_O_4_@g-C_3_N_4_ for the degradation of AMP.^66^ However, the degradation efficiency shown by NdFe_2_O_4_@g-C_3_N_4_ is higher than 95%, with a remarkable regeneration capacity for reuse. Capacity exhibited by NdFe_2_O_4_@g-C_3_N_4_ towards AMP compared better than studies on La/Cu/Zr trimetallic,^67^ FeSi@MN^68^ and ZnO/polyaniline nanocomposite.^69^ NdFe_2_O_4_@g-C_3_N_4_ has demonstrated the potential to remove CIP and AMP from an aqueous solution. The ease of synthesis and application of NdFe_2_O_4_@g-C_3_N_4_ as a photocatalyst gives it an additional advantage as a resource catalyst with potential in water treatment.

**Table 1:** Comparison of the photodegradation of CIP and AMP by NdFe_2_O_4_@g-C_3_N_4_ with other photocatalysts in literature^b^

Material	Antibiotic	DE (%)	LIS	AC (g L^−1^)	Conc (mg L^−1^)	Stability (%)	References
BiVO_4_	CIP	75.00	Visible light	1.00	10.00	—a	61
Cu_2_O/MoS_2_/rGO	CIP	55.00	150 W halogen lamp	0.30	10.00	—a	62
TiO_2_ on glass	CIP	92.00	6 W UV-C lamp	1.00	5.00	—a	70
ZnO	CIP	93.00	8 W Hg fluorescent	0.50	5.00	—a	71
ZnO	CIP	100.00	9 W Hg UV lamp	0.15	10.00	—a	65
TiO_2_/SnO_2_ composite	CIP	98.00	UVc lamps (35 W)	0.05	5.00	67.50 (3rd cycle)	63
*x*%BO/LNTO	CIP	100.00	300 W Xe lamp	2.00	10.00	95.00 (5th cycle)	72
R_2_-Cu_2_O	CIP	94.60	Metal halide lamp	1.50	20.00	—	73
ZnO/CD NCs	CIP	98.00	Sunlight	0.60	12.00	94.00 (5th cycle)	53
CeO_2_/g-C_3_N_4_	CIP	96.30	Visible light	1.00	10.00	90.40 (6th cycle)	64
MWCNTs-CuNiFe_2_O_4_	AMP	100.00	36 W UV	0.50	25.00	93.72 (8th cycle)	66
Ru/WO_3_/ZrO_2_	AMP	96.00	150 W Xe lamp	1.00	50.00	92.00 (2nd cycle)	74
La/Cu/Zr trimetallic	AMP	86.00	Sunlight	0.10	50.00	59.00 (6th cycle)	67
Ag-NP	AMP	96.50	Sunlight	0.17	10.00	—a	75
FeSi@MN	AMP	70.00	Sunlight	0.60	100.00	63.00 (4th cycle)	68
ZnO/polyaniline nanocomposite	AMP	41.00	Visible light	0.01	4.50	—a	69
NdFe_2_O_4_@g-C_3_N_4_	CIP	100.0	150 W Xe light	2.00	5.00	97.60 (15th cycle)	This study
	AMP	96.80		2.00	5.00	95.00 (15th cycle)	

a — = not reported.

b Degradation efficiency = DE, light illumination source = LIS, amount of catalyst = AC, Conc = concentration of antibiotic, Nickel–copper ferrite nanoparticles onto multi-walled carbon nanotubes = MWCNTs-CuNiFe_2_O_4_, FeSi@magnetic nanoparticle = FeSi@MN, ciprofloxacin = CIP, ampicillin = AMP, Ag-NP = silver nanoparticle *x*%BO/LNTO = La-doped NaTaO_3_ perovskites modified with a low quantity of Bi_2_O_3_.

## 4. Conclusion

Water pollution with antibiotics is a serious global challenge that requires immediate attention. Therefore, this study investigated the use of NdFe_2_O_4_ and NdFe_2_O_4_@g-C_3_N_4_ as photocatalysts for removing CIP and AMP from contaminated water systems. NdFe_2_O_4_ and NdFe_2_O_4_@g-C_3_N_4_ were synthesized *via* a simple chemical route. Their characterization revealed a crystallite size of 25.15 nm for NdFe_2_O_4_ and 28.49 nm for NdFe_2_O_4_@g-C_3_N_4_. The bandgap is 2.10 and 1.98 eV for NdFe_2_O_4_ and NdFe_2_O_4_@g-C_3_N_4_, respectively. The TEM images of NdFe_2_O_4_ and NdFe_2_O_4_@g-C_3_N_4_ revealed an average size of 14.10 nm and 18.23 nm, respectively. The surface morphology revealed from SEM images showed the surfaces of NdFe_2_O_4_ and NdFe_2_O_4_@g-C_3_N_4_ are heterogeneous, with irregular-sized particles suggesting agglomeration at the surfaces. The preliminary investigation of the degradation efficiency of NdFe_2_O_4_ and NdFe_2_O_4_@g-C_3_N_4_ revealed a better performance by NdFe_2_O_4_@g-C_3_N_4_ than NdFe_2_O_4_. NdFe_2_O_4_ had efficiencies of 78.45 ± 0.80 and 68.25 ± 0.60% for the photodegradation of CIP and AMP, respectively, while NdFe_2_O_4_@g-C_3_N_4_ has 100.00 ± 0.00 and 96.80 ± 0.80% for the degradation of CIP and AMP, respectively. Both photodegradation and adsorption were found to be taking place at the same time. NdFe_2_O_4_@g-C_3_N_4_ exhibited a stable regeneration capacity for the degradation of CIP and AMP even at the 15th cycle of treatment in a process that can be described by pseudo-first-order kinetic. The use of NdFe_2_O_4_@g-C_3_N_4_ in this study revealed its potential as a promising photocatalyst for removing CIP and AMP in an aqueous solution.

## Author contributions

Adewale Adewuyi: conceptualization, project design and execution, formal analysis, investigation, writing and review, validation, and editing. Rotimi A. Oderinde: conceptualization, editing, analysis, validation, and review.

## Conflicts of interest

The authors declare that they have no known competing financial interests or personal relationships that could have appeared to influence the work reported in this paper.

## Supplementary Material
